# Identification of the effector domain of biglycan that facilitates BMP-2 osteogenic function

**DOI:** 10.1038/s41598-018-25279-x

**Published:** 2018-05-04

**Authors:** Prapaporn Jongwattanapisan, Masahiko Terajima, Patricia A. Miguez, William Querido, Hideaki Nagaoka, Noriko Sumida, Elizabeth Grace Gurysh, Kristy M. Ainslie, Nancy Pleshko, Lalith Perera, Mitsuo Yamauchi

**Affiliations:** 1 Department of Veterinary Medicine, Faculty of Veterinary Science, Chulalongkorn University, Bangkok, 10330 Thailand; 2 Oral and Craniofacial Health Sciences, School of Dentistry, University of North Carolina at Chapel Hill, Chapel Hill, NC-27599 USA; 3 Department of Operative Dentistry, Oral and Craniofacial Health Sciences, School of Dentistry, University of North Carolina at Chapel Hill, Chapel Hill, NC-27599 USA; 4 Department of Bioengineering, Temple University, Philadelphia, PA-19122 USA; 5 Division of Pharmacoengineering and Molecular Pharmaceutics, Eshelman School of Pharmacy, University of North Carolina at Chapel Hill, Chapel Hill, NC-27599 USA; 6 Genome Integrity and Structural Biology Laboratory, National Institute of Environmental Health Sciences, National Institutes of Health, Research Triangle Park, NC-27709 USA

## Abstract

We have reported that recombinant biglycan (BGN) core protein accelerates bone formation *in vivo* by enhancing bone morphogenetic protein (BMP)-2 function. The purpose of the present study was to identify the specific domain (“effector”) within the BGN core protein that facilitates BMP-2 osteogenic function. Thus, we generated various recombinant and synthetic peptides corresponding to several domains of BGN, and tested their effects on BMP-2 functions *in vitro*. The results demonstrated that the leucine-rich repeats 2–3 domain (LRR2-3) of BGN significantly enhanced the BMP-2 induced Smad1/5/9 phosphorylation, osteogenic gene expression, and alkaline phosphatase activity in myogenic C2C12 cells. Furthermore, addition of LRR2-3 to osteoblastic MC3T3-E1 cells accelerated *in vitro* mineralization without compromising the quality of the mineral and matrix. These data indicate that LRR2-3 is, at least in part, responsible for BGN’s ability to enhance BMP-2 osteogenic function, and it could be useful for bone tissue regeneration.

## Introduction

Craniofacial bone defects are a major health threat to our society directly affecting the quality and length of human life. However, limitations still exist in the current treatments in terms of efficacy and costs. One of the key factors for bone regeneration is stimulation of osteogenesis via growth factors such as bone morphogenetic proteins (BMPs). BMPs were originally discovered by their ability to induce new bone and cartilage formation^1^ and the osteogenic function of BMP-2, -4 and -7 are all well documented^2^. Unfortunately, high doses of BMP are expensive and may lead to numerous potential adverse effects such as robust, ectopic, poorly organized bone formation as well as intense inflammation, soft tissue swelling, seroma, and cancer^3–7^. A critical barrier to progress in BMP-based bone regeneration is centered around a lack of understanding the optimal BMP dose to maximize osteogenic function while limiting potential side effects^8^.

Biglycan (BGN) is a ~150 kDa molecule composed of a ~45 kDa core protein and two glycosaminoglycan (GAG) chains. It was first identified in bone matrix^9^, but it is now clear that it is also present in non-skeletal tissues modulating various biological processes such as innate immunity, chemotaxis, angiogenesis, and growth factor regulation^10–12^. The role of BGN in osteogenesis was first demonstrated by the finding that BGN deficient mice developed an osteoporotic phenotype^13^. One of the potent mechanisms by which BGN facilitates osteogenesis is its ability to modulate critical factors such as BMPs, transforming growth factor-beta (TGF-β), and Wnt^14–17^.

In the past, we demonstrated that: 1. BGN directly binds BMP-2 and its receptors to accelerate osteoblast differentiation by positively modulating BMP-2/4 activity^14,18^, 2. The positive effect of BGN on BMP-2 function is derived from the core protein not the glycosaminoglycan (GAG) component^19^, and 3. When combined with low dose BMP-2, recombinant BGN, with no post-translational modifications, accelerates BMP-2 induced bone formation without compromising bone quality^20^. These findings led us to hypothesize that a specific peptide (“effector”) within the BGN core protein could be responsible for this function. Such effector then could be chemically synthesized and used in combination with low dose BMP-2 for bone regeneration.

To test this hypothesis, we generated several recombinant and synthetic peptides that correspond to specific domains of the BGN core protein, and tested each for their effects on the BMP-2 induced osteogenic function in order to identify the possible effector within the BGN core protein.

## Results

### Glutathione-S-Transferase (GST)-fused recombinant peptides

**Figure 1:**
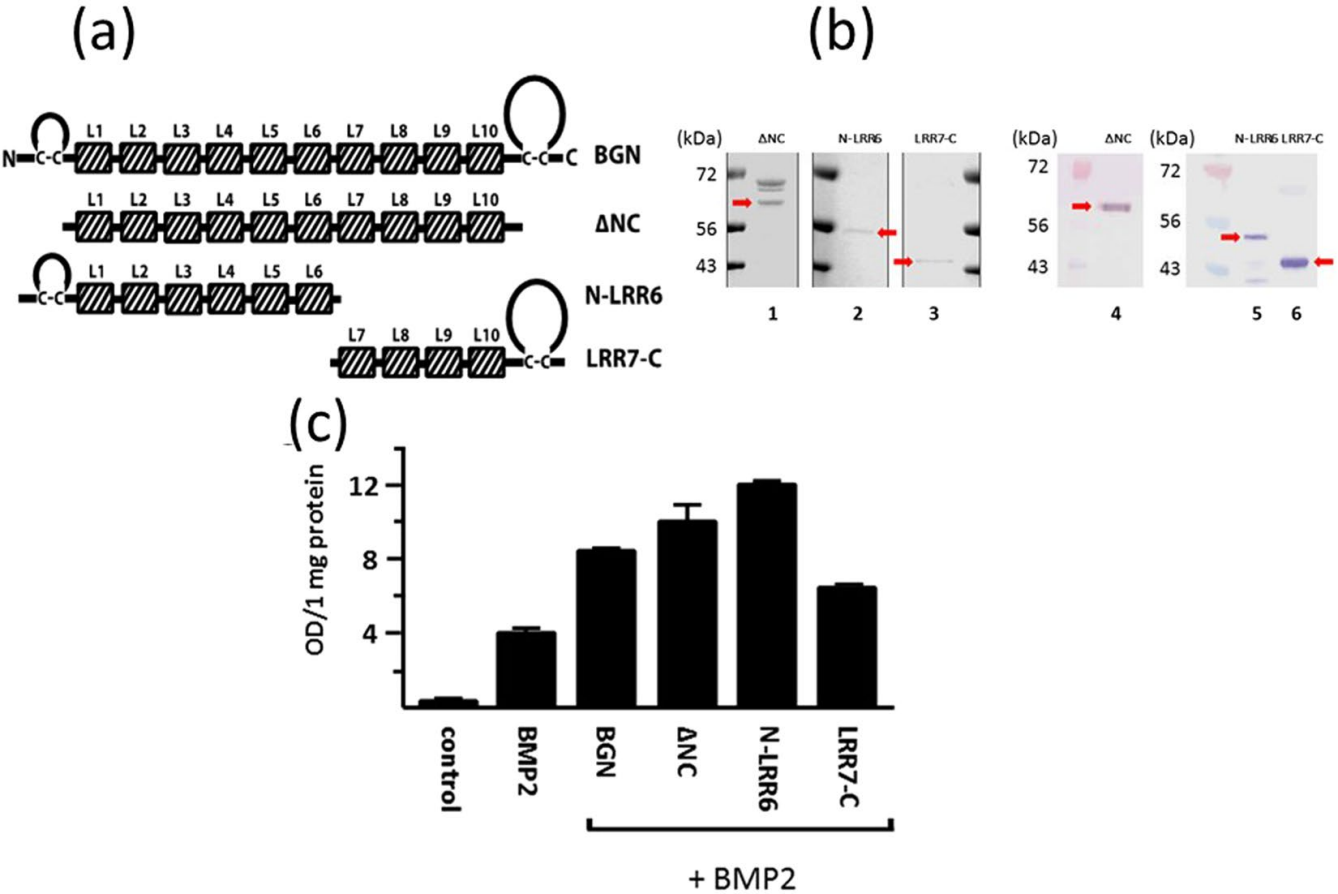
GST-fused BGN constructs and its effect on BMP-2 function. (**a**) Schematic drawings of biglycan (BGN) core protein and deletion constructs including core protein without N and C termini; ∆NC, N terminal to leucine rich repeat domain (LRR)6; N-LRR6, LRR7 to C terminus; LRR7-C. Each shaded box represents LRR (L in the illustration) of BGN. (**b**) The presence of glutathione-S-transferase (GST)-tagged BGN and deletion constructs were verified by SDS-PAGE stained with Coommassie brilliant blue (Left) and Western blot (WB) analyses with anti-GST antibody (right). The analyses showed the bands corresponding to ∆NC (~60 kDa) (lane 1), N-LRR6 (~50 kDa) (lane 2) and LRR7-C (~40 kDa) (lane 3) that were also immunopositive with anti-GST antibody (lanes 4–6, respectively) indicated by arrows. For full-length gel and Western blot images, see Supplemental Figure 1. (**c**) Alkaline phosphoatase (ALP) activity in C2C12 cells untreated (control), or treated with BMP-2 with or without BGN constructs. Note that BMP-2 induced ALP activity was further enhanced with the addition of BGN, ∆NC and N-LRR6. Values are shown as mean ± S.E. (n = 2).

The initial screening was done by using three, relatively large GST-fused BGN segments that were generated as reported^14^: (1) BGN core protein without N and C terminal cysteine loop regions (ΔNC), (2) N-terminus to leucine-rich repeat domain 6 (N-LRR6), and (3) LRR7 to C-terminus (LRR7-C) (Fig. 1a). The presence of these deletion constructs was confirmed by SDS-PAGE and Western blot (WB) analyses with an anti-GST antibody (Fig. 1b). Coomassie brilliant blue (CBB) stained gel and WB analysis showed the GST-positive bands of ΔNC at ~60 kDa (lanes 1 and 4, Fig. 1b), N-LRR6 at ~50 kDa (lanes 2 and 5, Fig. 1b) and LRR7-C at ~40 kDa (lanes 3 and 6, Fig. 1b), which are consistent with the expected molecular sizes with additional ~26 kDa GST tags. Though there were still some non-specific protein bands in these fractions, we proceeded to test which construct-containing fractions exhibit the activities. Each fraction was subjected to alkaline phosphatase (ALP) assay using C2C12 cells. This murine-derived myogenic cell line re-differentiates into osteoblastic cells upon BMP-2 treatment^21,22^, as such, it is widely used to evaluate the osteogenic function of BMP-2. The whole BGN-GST enhanced the BMP-2 induced ALP activity (Fig. 1c), thus confirming our previous finding^14,20^. Both ΔNC-GST and N-LRR6-GST fractions also showed this effect whereas LRR7-C fraction was not as effective as these three (Fig. 1c). However, we cannot rule out the possibility that the LRR7-C also contains the effector region as the ~34 kDa GST-positive contaminants in this fraction (Supplemental Figure S1) could have a negative effect on the activity. GST-BGN and GST alone showed no effect as reported^20^ (data not shown). These experiments were repeated three times and done in duplicate each time. The trend was consistent across repetitions, and Fig. 1c represents one of the replicates. When the groups were compared by JMP program (SAS, Cary, NC) using a two-tailed t-test at 0.05 significance level (n = 2/group), the ALP activities of ΔNC-GST + BMP, BGN-GST + BMP and N-LRR6-GST + BMP were significantly higher than BMP alone while LRR7-C-GST + BMP was not. These data suggest that the effector peptide most likely resides between LRR1 and LRR6.

### LRR2-3 peptide enhances BMP-2 osteogenic function

**Figure 2:**
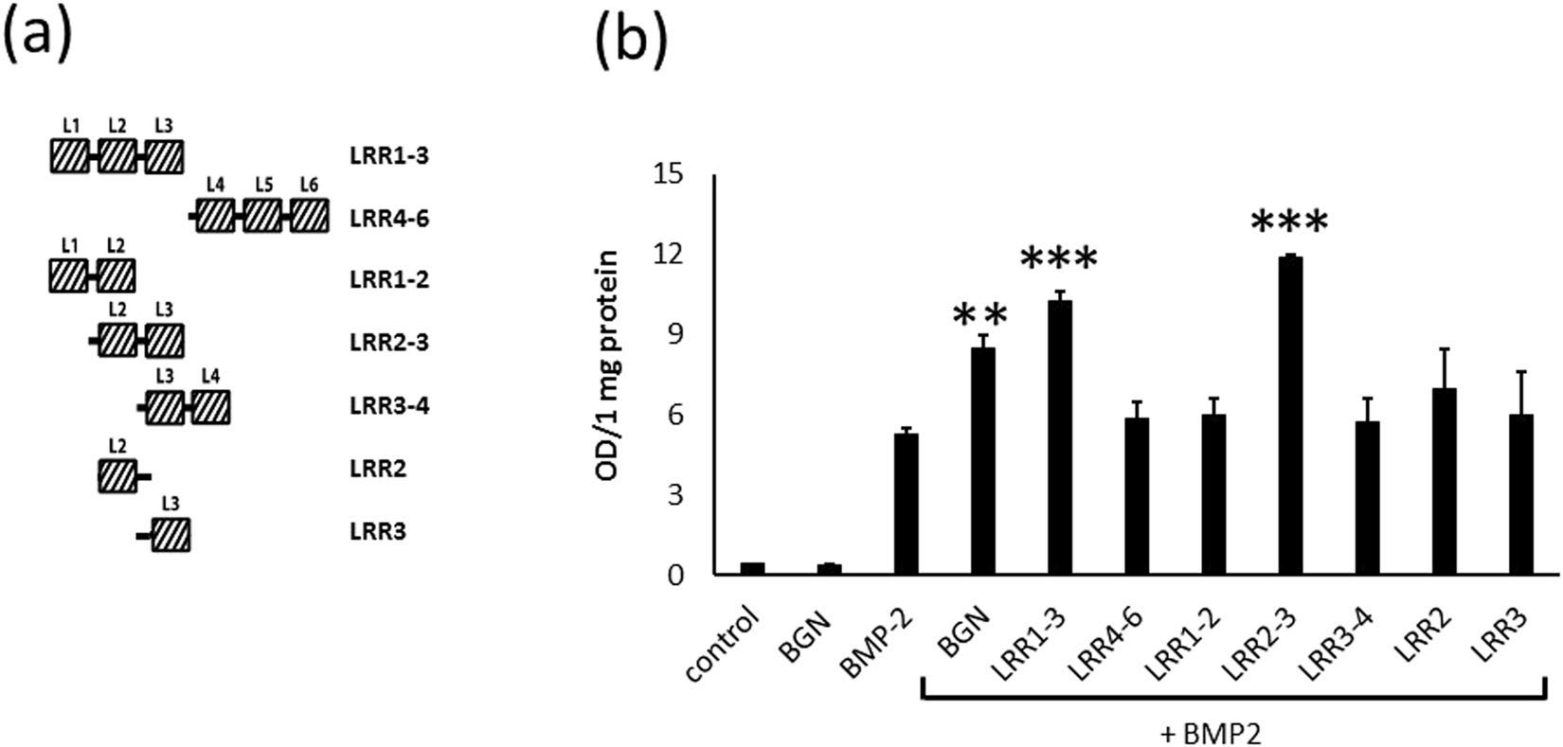
Synthetic peptides and its effect on ALP activity. (**a**) Schematic drawings of synthetic peptides derived from LRR 1–6 including LRR1-3, LRR4-6, LRR1-2, LRR2-3, LRR3-4, LRR2 (with a hinge) and LRR3 (with a hinge). (**b**) ALP activities in C2C12 cells. Note that the most significant effect was found in combination of BMP-2 with LRR2-3. Values are shown as mean ± S.E. (n = 3). ^**^p < 0.01, ^***^p < 0.001.

Thus, we synthesized several segments derived from LRR1-6 (Fig. 2a) and tested their effects on BMP-2 function using C2C12 cells as described above. BMP-2 treatment induced ALP activity while the human recombinant BGN (R&D Systems) alone showed no effect (Fig. 2b). The synthetic peptides corresponding to LRR1-3 and LRR4-6 (Fig. 2a) were first tested and the results showed that LRR1-3 significantly enhanced the BMP-2 activity (p < 0.001) like recombinant BGN but not LRR4-6 (p = 0.4930) (Fig. 2b). On the basis of these results, we further synthesized the peptides LRR1-2, 2-3 and 3-4 (Fig. 2a) and tested their effects. The results demonstrated that LRR2-3 most enhanced the BMP-2 induced ALP activity (p < 0.001) (Fig. 2b) while LRR1-2 or LRR3-4 showed no significant effect. Interestingly, the effect of LRR2-3 was even higher than that of LRR1-3 (p < 0.05) (Fig. 2b). In an effort to further identify the effector domain within the LRR2-3 peptide, LRR2-hinge (hinge: peptide between LRR2 and 3) and hinge-LRR3 were also synthesized and tested. However, neither one of these shorter peptides exerted significant effect (Fig. 2b) suggesting that the peptides longer than these may be required for this function (see Molecular Dynamic Simulation section).

### LRR2-3 enhances BMP-2 induced osteogenic gene expression in C2C12 cells

**Figure 3:**
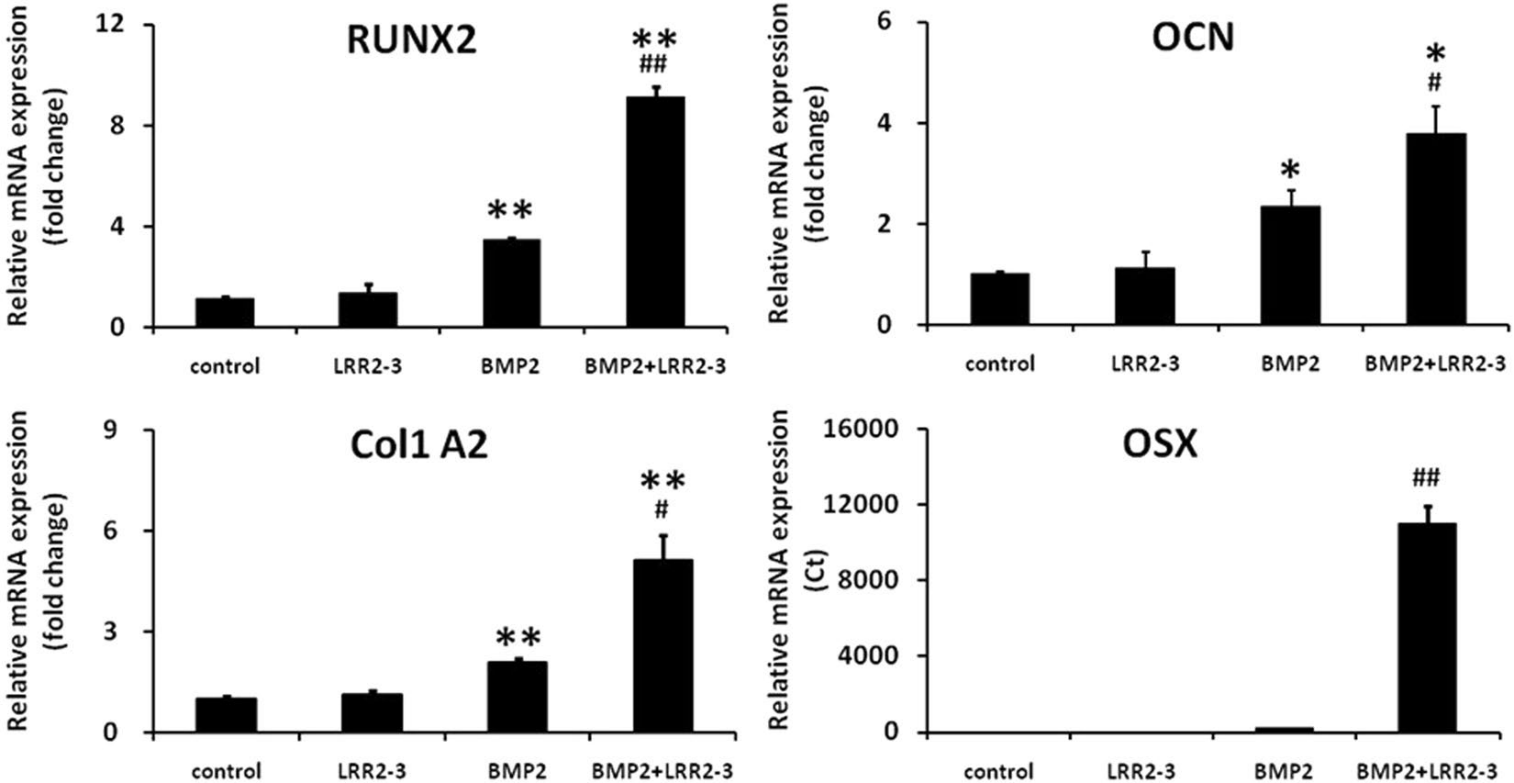
Effect of LRR2-3 on BMP-2 induced osteogenic gene expression in C2C12 cells. Runx2: runt-related transcription factor 2, Col1A2: type I collagen alpha 2, OCN: osteocalcin, OSX: osterix. Values are shown as mean ± S.E. (n = 3). Since the expression of OSX was detected only in BMP-2 alone and BMP-2 with LRR2-3, the expression is shown in Ct values. ^*^p < 0.05, ^**^p < 0.01 between control and BMP2 alone or BMP2 with LRR2-3; ^#^*p* < 0.05, ^##^*p* < 0.01 between BMP2 alone and BMP2 with LRR2-3.

Next, we investigated whether or not the addition of the synthetic peptide LRR2-3 to BMP-2 changes the osteogenic and myogenic gene expression in C2C12 cells (Fig. 3). LRR2-3 alone did not change any of the marker genes tested after a 2-day exposure. Treatment with BMP-2 alone significantly increased the expression of osteogenic genes Runx2, Col1A2, OCN, and OSX (p < 0.01). Combination treatment with LRR2-3 and BMP-2 led to ~2 fold further enhancement (p < 0.05) of osteogenic gene expression levels for Runx2, Col1A2, and OCN. Of note, the effect on OSX expression was the most significant of all the genes examined. It was hardly detected in control and LRR2-3 alone groups, and, upon the BMP-2 treatment, it was detected at a higher, albeit low, level. However, with the addition of LRR2-3 to BMP-2, it showed a 300–400-fold increase in comparison to BMP-2 alone. The expression of Myod1, a myogenic gene, in C2C12 cells tended to diminish when treated with BMP-2 alone or BMP-2 with LRR2-3 though this difference was not statistically significant (data not shown).

### Effect of LRR2-3 on BMP-2 induced signaling

**Figure 4:**
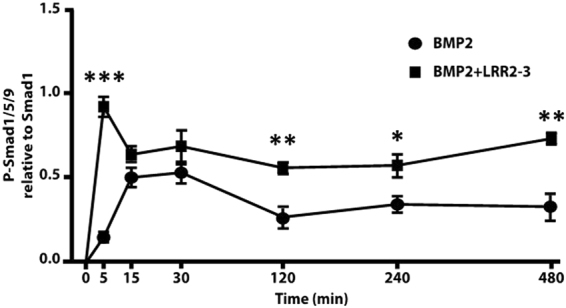
BMP-2 induced Smad phosphorylation. C2C12 cells were treated with 75 ng/mL of BMP-2 alone (closed circle) or BMP-2 with the addition of LRR2-3 (closed square) at various time points. Cell lysates were analyzed for Smad phosphorylation by normalizing phosphorylation (P)-Smad1/5/9 to Smad1. Three independent experiments were performed and values are shown as mean ± S.E. (n = 3). ^***^p < 0.001, ^**^p < 0.01, ^*^p < 0.05.

The effect of LRR2-3 on the BMP-2 induced signaling was assessed by Smad 1/5/8 phosphorylation in C2C12 cells. When BMP-2 was added to the cells, Smad phosphorylation occurred during the first 30 min, decreased at 120 min and remained that level thereafter (Fig. 4 and Supplemental Figure S2). With the addition of LRR2-3 to BMP-2, the phosphorylation occurred more rapidly, peaking at 5 min and achieved a higher level (~5 fold increase compared to BMP-2 alone). Although phosphorylation decreased at 15 min, combination treatment with BMP2 and LRR2-3 led to higher sustained levels of phosphorylation for up to 480 min (Fig. 4 and Supplemental Figure S2). The data indicate that LRR2-3 enhances and sustains the BMP-2 signaling at least in part through the canonical signaling pathway. We previously reported that GST-BGN could enhance^20^ and sustain^14^ the BMP-2 transduced Smad phosphorylation. Thus, the data suggests that LRR2-3 can exert a similar effect on BMP-2 signaling induced by whole BGN core protein.

### *In Vitro* Mineralization Assay

**Figure 5:**
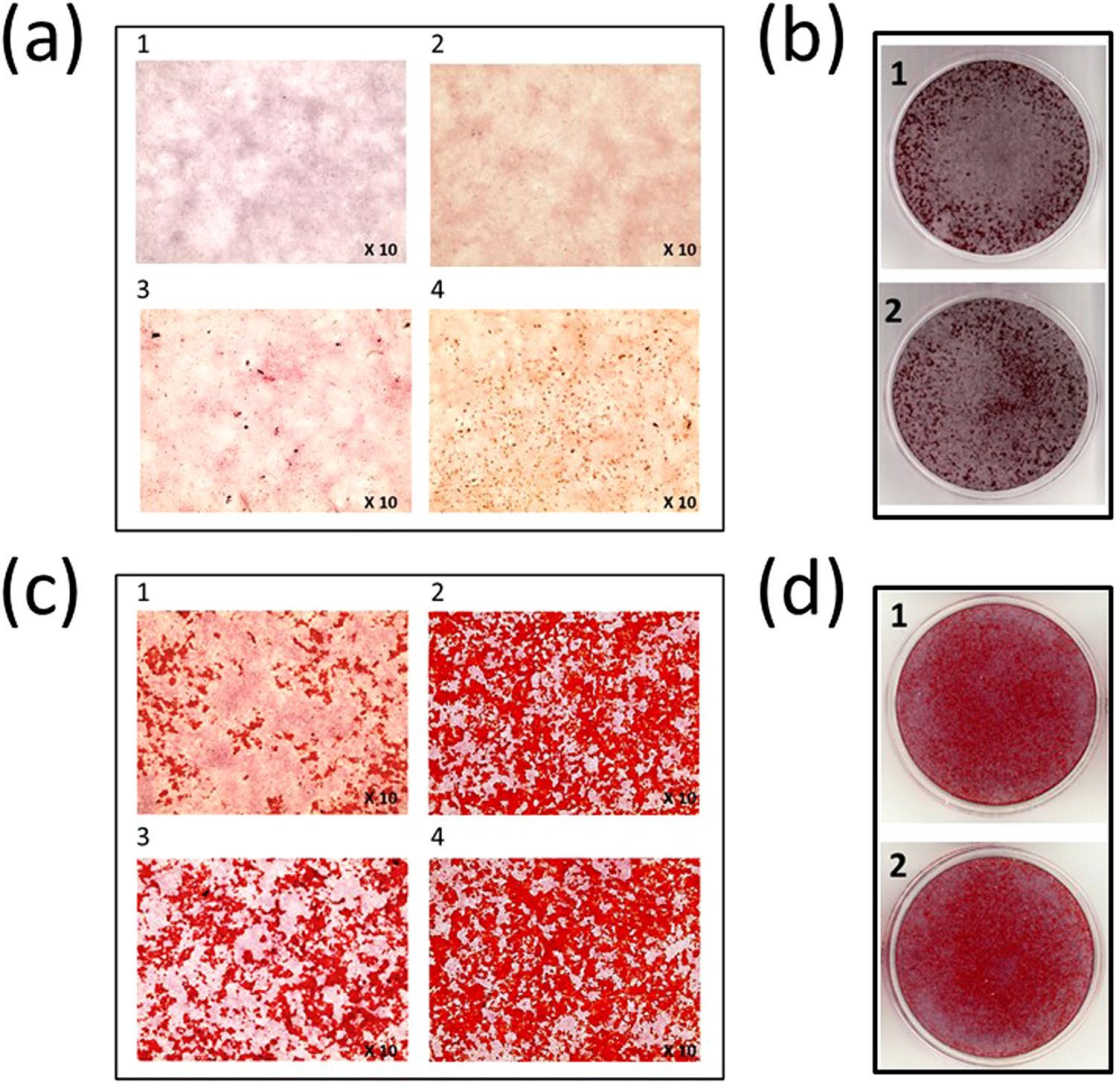
*In vitro* mineralization assay. MC3T3-E1 (MC) cells were cultured and stained Alizarin Red S (ARS). Upper panel. MC cells at day 11 (**a**-1) and 14 (**a**-2) and those treated with LRR2-3 at the same time points (**a**-3 and a-4). (**b**) Scanned cultures at day 28 (**b**-1: MC cells, **b**-2 MC cells treated with LRR2-3). Lower panel. MC cells treated with BMP-2 at day 11 (c-1) and 14 **(c**-2) and those treated with BMP-2 and LRR2-3 at the same time points (**c**-3 and **c**-4). (**d**) Scanned cultures at day 14 (**d**-1: BMP-2, **d**-2: BMP-2 and LRR2-3).

To examine the effect of LRR2-3 on the timing of mineralization, MC3T3-E1 (MC) cells were cultured in mineralized medium with or without LRR2-3 and stained with Alizarin red S (Fig. 5a). In another set of experiments, cells were treated with BMP-2 with or without LRR2-3 and mineralization was assessed (Fig. 5c). Without any treatment, MC cells showed no mineralized nodules at day 11 (Fig. 5a-1) and started showing some at day 14 (Fig. 5a-2). However, with the addition of LRR2-3 to the cells, the nodule formation was evident at day 11 (Fig. 5a-3) and the nodules were further increased at day 14 (Fig. 5a-4). At 4 weeks, MC cell cultures with or without LRR2-3 addition were well mineralized and there were no significant differences between the two groups (Figs 5b-1 and 2). These data demonstrates that LRR2-3 alone can accelerate *in vitro* mineralization in MC cells in which BMP-2 is endogenously expressed^23^. When MC cells were treated with BMP-2, mineralized nodules were rapidly and extensively formed even at day 11 (Fig. 5c-1). Combination treatment with LRR2-3 and BMP-2 led to slightly faster nodule formation than those treated with BMP-2 alone. (Figs 5c-1 and -3). At day 14, the cultures were fully mineralized in both treatment groups (Figs 5c-2 and -4, and 5d-1 and 2).

### Fourier Transform Infrared Spectroscopy (FTIR) analysis

**Figure 6:**
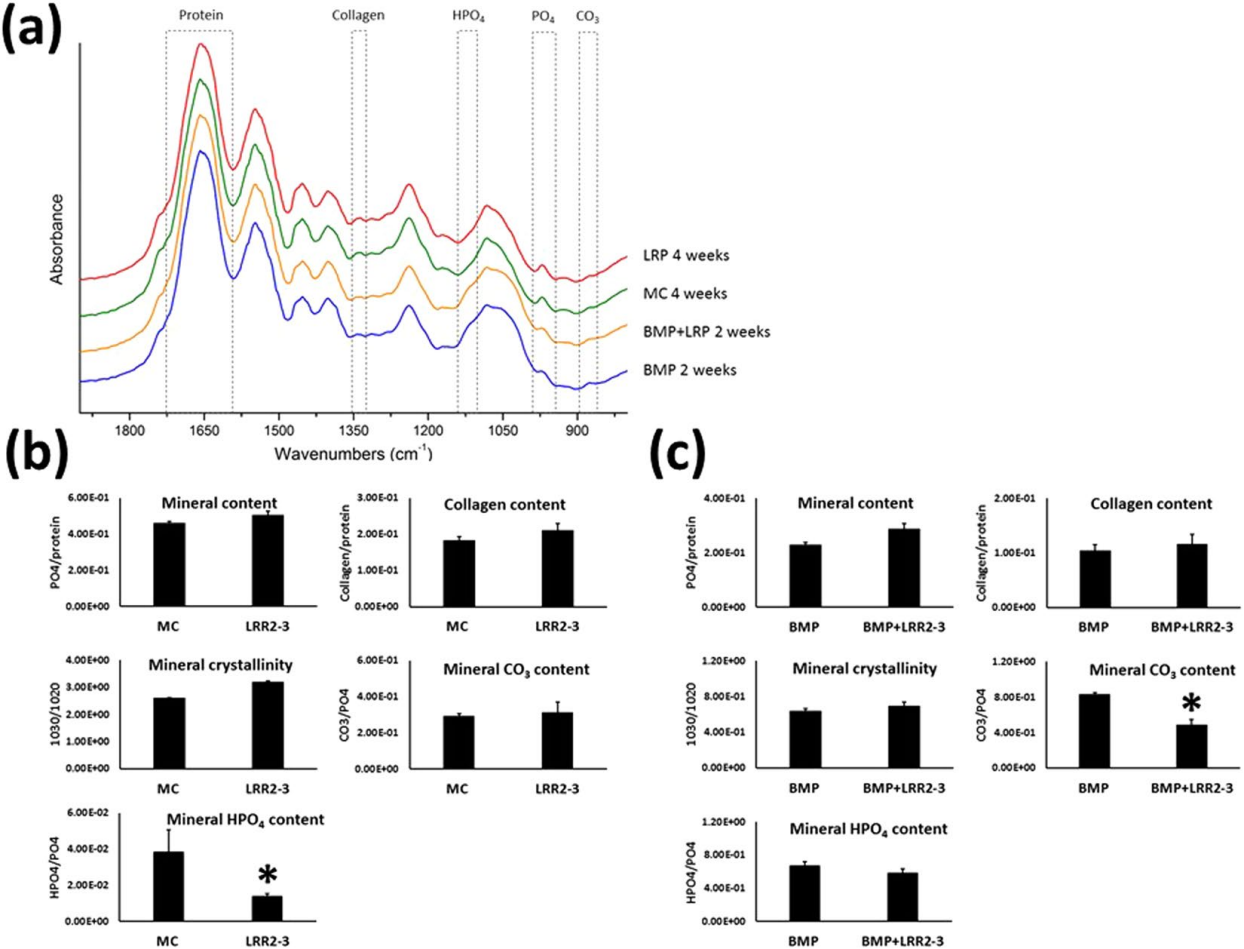
Fourier transform infrared (FTIR) spectroscopy analysis. (**a**) FTIR spectra of the cell culture samples, highlighting the regions of interest: amide I absorbance band for protein, collagen absorbance, the ν_1_ PO_4_ absorbance band reflecting mineral, the CO_3_ absorbance band reflecting carbonate incorporated into the mineral, and the HPO_4_ band reflecting acid phosphate incorporated into mineral. Second derivative spectra were used to narrow the broad absorbance peaks, and the peak intensities reported are proportional to the amount of the protein and mineral components present in the cultures. The mineral crystallinity was also investigated based on the ratio of second derivative peaks in the ν_3_ PO_4_ band at ~1030 and 1020 cm^−1^. (**b**) untreated MC cells or LRR2-3 alone (day 28), (**c**) BMP-2 alone or addition of LRR2-3 to BMP-2 (day 14);. Treatment with LRR2-3 was shown to preserve the development and features of the mineral and matrix formed in the cell cultures, but also resulted in lower levels of non-stoichiometric components of the mineral (CO_3_, HPO_4_). Samples were analyzed in triplicate (n = 3) and are shown as mean ± S.E. ^*^p < 0.05.

Key features of the mineral and matrix formed in the cell cultures with or without LRR2-3 were analyzed using FTIR (Fig. 6a). MC cell cultures and those treated with LRR2-3 were analyzed at 4 weeks when mineralized nodules were well formed (Fig. 5b). The addition of LRR2-3 to MC cells did not lead to changes in the mineral content, collagen content, mineral crystallinity, or mineral CO_3_ content, but did lead to significantly lower mineral HPO_4_ content (Fig. 6b). MC cell cultures treated with BMP-2 alone or with the addition of LRR2-3 were analyzed at 2 weeks when nodules were already excessively deposited in both groups (Fig. 5d). No differences were observed between the two groups in the mineral content, collagen content, mineral crystallinity and mineral HPO_4_ content (Fig. 6c). However, a significantly lower mineral CO_3_ content was observed in the cultures treated with BMP-2 and LRR2-3 compared to those treated with BMP-2 alone. Compared to the mineralized cultures without BMP-2 at 4 weeks, BMP-2 addition at 2 weeks resulted in lower mineral crystallinity and higher amounts of HPO_4_ and carbonate, which is indicative of the formation of an immature mineral phase.

### Molecular Dynamic Simulations

**Figure 7:**
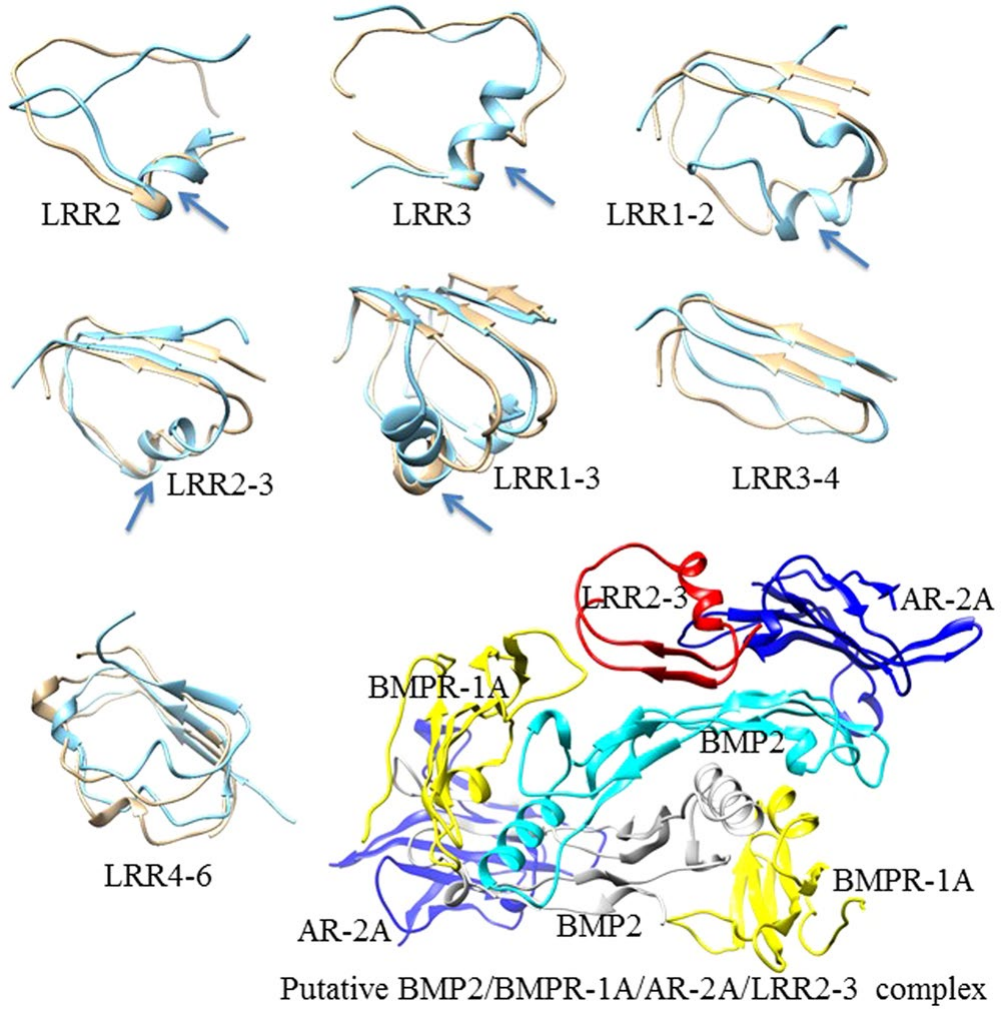
Molecular dynamic simulations. Representative snapshots of peptides from solution structure evaluations (in cyan). Also shown are the starting segments from X-ray crystal structures (in tan). An emergence of a helix in each structure during dynamics is indicated with the arrow in LRR2 (with a hinge), LRR3 (with a hinge), LRR1-2, LRR1-3, and LRR2-3 peptides. A putative model showing LRR2-3 interactions with the BMP2/BMPR-1A/AR-2A dimer system (LRR2-3 in red; BMP2 dimer in cyan and gray; BMPR type 1 A in yellow; activing receptor type 2 A in blue).

In an attempt to structurally characterize various LRR peptides that were tested for the activity, i.e. LRR1-2, 2 (with a hinge), 3 (with a hinge), 2–3, 1–3, 3–4 and 4–6, we have evaluated solution structures of these peptides after lengthy molecular dynamics (MD) simulations of over 120 ns for each peptide. Since all initial peptide conformations were originally based on the X-ray crystal structure of the full-length BGN dimer structure^24^, we first studied what structural changes these short peptides would undergo in the absence of their surrounding residues. Dynamic nature and the stability of these peptides can be analyzed using the root mean square deviations (RMSD) of the backbone atoms from the final structures calculated with reference to the initial x-ray structure and the RMSD values are given in the Supplemental Table S2. In addition, Supplemental Table S2 includes the total charge with total number of positively and negatively charged residues since these charged residues that are solvent exposed, in general, tend to occupy on the surface that is available for BMP (or BMP receptor) interactions. The snapshots of the final structures of each peptide are given in Fig. 7. For comparison, we have also shown the backbone structures of the aligned initial x-ray structures.

An emergence of a helical segment is observed (highlighted with arrows in Fig. 7) in some structures from our simulations when compared them with the starting models from X-ray crystals. The segment of residues that are common to both LRR2-hinge and hinge-LRR3 peptides with sequence EKAFSPLR in the hinge region participated in the formation of the helix (with an additional lysine residue present in the helix of LRR3). However, these individual peptides LRR2-hinge and hinge-LRR3 exhibit the largest averaged RMSD values reported from our MD simulations (Supplementary Table S2) indicating that these two peptides undergo the largest structural changes compared to their folds in the segment from the full-length BGN. Compared to this highly dynamic behavior observed in LRR2-hinge and hinge-LRR3 peptides, for LRR2-3 which facilitated the greatest enhancement of BMP-2 osteogenic function in experiments, our molecular dynamics trajectory calculations yielded the smallest averaged RMSD value (Supplementary Table S2) indicating the helical segment in the hinge region between LRR2 and LRR3 provides extra stability to the observed fold of this peptide. This added stability provided by the helix in the hinge region in combination with the extra exposure and the freedom of the residues towards protein interactions (when compared to the corresponding segment in the full-length BGN) possibly makes LRR2-3 a better peptide with significantly improved effector function. In LRR2-3 and LRR1-3, this helix is folded in such a way that their non-polar residues are clustered internally. In the LRR1-2 peptide, a segment with a different amino acid composition (KDFKG) refolded to yield a helix during the course of MD simulations (indicated by an arrow in Fig. 7), but the conformation freedom of this peptide appears much higher and the resulting structure is more dynamic (large RMSD shown in Supplemental Table S2). Although the peptide LRR1-3 also has this segment fully evolved to be helical, it is evident that this segment is not fully exposed for binding as in the case of LRR2-3, because of the self-interactions with the neighboring helix (Fig. 7). Interestingly, in the peptide LRR4-6 a small helix emerges during the MD run in the peptide segment containing residues KGVFSGLR. In fact, there is a high sequence homology of this amino acid segment to the segment EKAFSPLR of peptides LRR2-3 and LRR1-3 that displayed a helical character appeared during dynamics. The emergence of the helix may be playing a role in the slight activity associated with the peptide LRR4-6. A putative model showing LRR2-3 interactions with the BMP2/BMPR-1A/AR-2A dimer system is created using the equilibrated LRR2-3 solution structure and the X-ray crystal structure of the BMP2/BMPR-1A/AR-2A complex and is shown in Fig. 7.

## Discussion

In this study, we have identified the specific region of BGN core protein, LRR2-3, as the potential effector peptide that facilitates BMP-2 osteogenic function. The synthetic LRR2-3 peptide enhanced BMP-2 induced Smad signaling, osteogenic gene expression, and alkaline phosphatase activity. Furthermore, addition of LRR2-3 to MC cells showed that the effector peptide is capable of enhancing BMP-2 osteogenic function without compromising the quality of the mineral and matrix. Exogenous addition of BMP-2 with or without LRR2-3 to MC cells induced robust mineralization even at early stage of cultures, but with poorer mineral quality. This mineral could be a very immature and non-stoichiometric phase, formed in a above physiological rate by “excessive” BMP-2. This would be in line to the robust but poorly organized bone formation observed in a rat mandible defect with high dose BMP-2^20^. These data indicate that LRR2-3 of BGN combined with low dose BMP-2 is capable of enhancing BMP-2 osteogenic function without compromising the mineralization process.

It has been shown that *Bgn*-deficient osteoblast had poor ability to respond to BMP-4 stimulation and eventually led to a defect in osteoblast differentiation indicating that BGN controls osteoblast differentiation by modulating BMP function^16^. By employing *in vitro* gain- and loss-of-function approaches, we also showed that BGN positively modulates osteoblast differentiation possibly by regulating BMP function^18^. Interestingly, significant “direct” binding of BGN was observed with BMP-2 and its type I receptor, ALK6^14^. The effect of BGN on BMP-4 thus could be more complicated than that of BMP-2 by involving other molecules. Our *in vivo* study showed that GST-fused BGN core protein in combination with low dose BMP-2 accelerated bone formation in a rat mandibular defect model without compromising the newly formed bone quality. In the same model, high dose BMP-2 induced robust, but poorly organized ectopic bone formation with an inflammatory response^20^. These findings led us to hypothesize that the specific effector peptides within the BGN core protein facilitates BMP-2-induced osteogenesis. This could be translationally significant since use of such effector could lower the dosage of BMP-2 to form new bone of high quality, thus, it would be cost effective and could minimize adverse side effects associated with high dose BMP-2.

The hallmark of bone is its mineralized matrix. Bone strength and resistance to fracture is not only determined by the amount and density of bone, but also by the quality of the mineralized tissue, which includes its microarchitecture and properties of the organic and mineral components^25,26^. The mineral found in bone is a poorly crystalline apatite that contains several non-stoichiometric features, such as CO_3_ and HPO_4_ impurities^27^. With age, the apatite crystals become more stoichiometric and the crystallinity of the mineral increases^28^. A decrease in CO_3_ and HPO_4_ has previously been described with maturation of bone tissue^29^. Here, our results demonstrate that the LRR2-3 effector peptide preserves a normal bone-like matrix quality, without affecting collagen formation, degree of matrix mineralization and mineral crystallinity. Moreover, the lower levels of non-stoichiometric components of the mineral (CO_3_, HPO_4_) seen in cultures in the presence of LRR2-3 may suggest that the effector could play a role in promoting matrix development.

The molecular mechanism by which LRR2-3 facilitates BMP-2 function is not clear at this point, but MD simulations may provide some structural insight. The net charge of the peptide seems to be important as the majority of charged residues are located on the surface that likely interact with BMP, BMPR or BMP/BMPR complex. It is notable that LRR2-3, the most effective peptide, contains the largest positive charge of all the peptides (Supplemental Table S2). Interestingly, in LRR2-3, residues EKAFSPLR which resides in the “hinge” region between LRR2 and 3, refolded to yield a helix during the course of MD simulation. Though the peptide LRR1-3 contains the same segment, possibly due to some interactions with LRR1, it has not fully evolved to be a tighter helical segment. We have created a model system with BMP-2 dimer interacting with BMP receptor type IA and activin receptor type 2 A based on available X-ray crystal structures^30^. We note here that the system containing each BMP-2 and its associated receptors possesses a net negative charge of 12e. This BMP2/BMPR-type IA and activing type 2 A dimer system was used in the preliminary docking study with LRR2-3. This docking complex, though a preliminary working model, indicates that the LRR2-3 sits on a surface interacting with BMP-2 as well as its partner receptors. In addition, the location of LRR2-3 on the BPM-2 surface might reduce the mobility of BMP-2 so that it will potentially help stabilize the BMP-2 dimer interactions. Thus, LRR2-3 may function as a bridge molecule to enhance the stability of BMP-2 interaction with its receptor partners. Further structural studies utilizing NMR and X-ray crystallography may provide more definitive structural information critical for the interaction between the effector, BMP-2, and its receptors.

The therapeutic benefit of BMPs combined with scaffolds for bone formation is very promising but is hampered by scaffolds’ rapid degradation *in situ*, high costs, and the adverse effects associated with a high dose of BMP-2. The current data indicate that the effector in BGN facilitating the BMP-2 osteogenic function resides in the LRR2-3 domain and the synthetic peptide could effectively promote osteogenesis in combination with low dose BMP-2. Further studies could investigate the effect of controlled release of the effector peptide in combination with BMP-2 from a biodegradable polymeric scaffold on osteogenesis *in vivo*. It is possible that sustained delivery of the effector peptide could facilitate BMP-2 function and while minimizing the cost of BMP-2 treatment and adverse effects associated with high dose BMP-2.

## Materials and Methods

### Cell culture and synthetic peptides

C2C12 myoblastic cells were obtained from American Type Culture Collection (ATCC; CRL-1772) and were maintained according to the manufacturer’s instructions. Briefly, cells were grown in Dulbecco’s Modified Eagle Medium (DMEM, Gibco®, NY) containing a high concentration of glucose (4.5 g/L) and supplemented with 15% fetal bovine serum (FBS), 100 U/mL of penicillin G sodium, and 100 μg/mL of streptomycin sulfate. Cells were maintained at 37 °C in a humidified 5% CO_2_ incubator and the medium was changed every two days.

### Synthetic peptides

The peptides LRR1-3, LRR4-6, LRR1-2, LRR2-3, LRR3-4, LRR2-hinge and hinge-LRR3 were commercially synthesized (Peptide 2.0 Inc., VA) and the purity of each peptide was greater than 90% based on high performance liquid chromatographic analysis. The peptide sequences are shown in Table 1.

**Table 1:** Amino acid sequence of synthetic peptides for each core protein LRR.

LRR peptide	Sequence
LRR1-3	TLLDLQNNDISELRKDDFKGLQHLYALVLVNNKISKIHEKAFSPLRKLQKLYISKNHL
LRR4-6	SLVELRIHDNRIRKVPKGVFSGLRNMNCIEMGGNPLENSGFEPGAFDGLKLNYLRISEAKL
LRR1-2	TLLDLQNNDISELRKDDFKGLQHLYALVLVNNKI
LRR2-3	HLYALVLVNNKISKIHEKAFSPLRKLQKLYISKNHL
LRR3-4	KLQKLYISKNHLVEIPPNLPSSLVELRIHDNRI
LRR2-hinge*	HLYALVLVNNKI-SKIHEKAFSPLR
hinge*-LRR3	SKIHEKAFSPLR-KLQKLYISKNHL

^*^Hinge: amino acid sequence between LRR2 and 3.

### Glutathione-S-transferase (GST)-fused BGN constructs

Full length BGN core protein with GST (GST-BGN) was generated as described^14,20^. In the same manner, three GST-tagged BGN deletion constructs were generated: BGN core protein without N and C termini where cysteine-loops are present (ΔNC), N-terminus to LRR6 (N-LRR6), and LRR7 to C-terminus (LRR7-C). Briefly, the PCR products were inserted into pGEX4T-1 vector (Amersham Biosciences, NJ), sequenced and the pGEX4T-1- mutant *Bgn* constructs were obtained. The plasmid were transformed into BL21-CodonPlus bacterial strain (Stratagene, CA) and the protein/peptide synthesis was induced by isopropyl-D-1-thiogalactopyranoside (IPTG, Fisher Scientific, MA). The bacteria were cultured at 20 °C overnight, centrifuged and lysed in phosphate buffed saline (PBS) containing 1% Triton X-100. The GST-protein/peptides were purified by molecular sieve chromatography using a Superdex 200 (GE Healthcare, WI) column in 2 M guanidine-HCl, pH 7.5. The eluted proteins were dialyzed against distilled water for 3 days and lyophilized. The peptide concentrations were determined by the DC protein assay kit (Bio-rad, CA).

### Alkaline Phosphatase (ALP) Activity

C2C12 cells were plated onto 35 mm culture dishes at a concentration of 50×10^3^ cells/dish and grown over night. On the following day, the medium was changed to DMEM supplemented with 5% FBS, 100 U/mL of penicillin G sodium, and 100 μg/mL of streptomycin sulfate. Cells were treated with 4 μg/mL of recombinant BGN core protein (R&D systems, MN), GST-fused peptides or synthetic peptides with or without 75 ng/mL of rhBMP-2 (R&D systems, MN). Three concentrations of BMP2, i.e. 300 ng/mL^21^ or lower (150, 75 ng/mL) combined with BGN were tested. Since the lowest concentration (75ng/mL) was sufficient to observe the BMP2 activity and the BGN effect, this concentration was used for this study (data not shown). The medium was changed at day 2 and, at day 4 cells were washed twice with cold PBS and lysed with buffer containing Triton X-100. The supernatants were subjected to ALP activity assay by using an alkaline phosphatase kit (Sigma Diagnostics, MI) according to the manufacturer’s instructions.

### Quantitative Real Time RT-PCR

C2C12 cells were plated onto 35 mm cultures dishes at a concentration of 75 × 10^3^ cells/dish. On the following day, the medium was change to DMEM containing 5% FBS, 100 U/mL of penicillin G sodium, and 100 μg/mL of streptomycin sulfate supplemented with 75 ng/mL of rhBMP-2 combined with or without 4 μg/mL of LRR2-3 for 48 hours. Total RNA was prepared using TRIzol reagent (Invitrogen, MA) and purity of the total RNA was evaluated by a NanoDrop 2000cspectrophotometer (Thermo Scientific, MA) at 260 and 280 nm. Two microgram of total RNA was reverse-transcribed to cDNA by using Omniscript RT Kit (Qiagen, MD) according to the manufacturer’s protocol. The primers-probes of osteogenic and myogenic markers used for real time PCR were as follows (Supplemental Table S1); core binding factor 1/runt-related transcription factor 2 (*Cbfa1/Runx2*, ABI assay number: Mm00501578_m1); type I collagen alpha 2 chain (*Col1A2*, Mm00483888_m1); osteocalcin (*Ocn*, Mm01741771_g1) osterix (*Osx*, Mm00504574_m1); myogenic differentiation 1 (*Myod1*, Mm00440387_m1). By using the ABI Prism 7000 Sequence detection system (Applied Biosystems), real time PCR was analyzed in triplicate. The relative expression of the studied genes over glyceraldehyde-3-phosphate dehydrogenase (*Gapdh*, ABI assay number: 4308313) was calculated from the threshold cycle (C_T_) by using 2^−ΔΔCT^ method as described previously^31^.

### Smad Phosphorylation Assay

C2C12 cells were plated onto 35 mm culture dishes at a concentration of 1 × 10^5^ cells/dish. On the next day, cells were treated with 75 ng/mL of rhBMP-2 combined with 4 μg/mL of LRR2-3 synthetic peptide. At 5, 15, 30, 120, 240 and 480 min, cells were washed with RIPA buffer and centrifuged. Twenty microliters of the supernatant was mixed with 3 × SDS sample buffer, applied to 4–12% SDS-PAGE followed by Western blot analyses with anti-phospho-Smad1/5/9 antibody (Cell Signaling Technology, MA), or anti-Smad1 antibody (Cell Signaling Technology, MA). Signals were detected by immunoreactivity and were analyzed by ImageQuant^TM^ LAS 4000 (GE Healthcare, IL). The levels of Smad1/5/9 phosphorylation was calculated relative to total Smad1 level of each sample.

### *In Vitro* Mineralization Assay

MC cells were plated at a density of 2 × 10^5^ cells per 35 mm dish and cultured in α-minimum essential medium containing 10% FBS, 100 U/mL penicillin, and 100 μg/mL streptomycin. Upon confluence, cells were maintained in the mineralization medium containing 50 μg/mL ascorbic acid and 2 mM β-glycerophosphate with or without 8 μg/mL of LRR2-3 synthetic peptide and cultured for up to 2 weeks. At days 11 and 14, the cell/matrix layer from each sample was washed with PBS, fixed with 100% methanol, and stained with 1% Alizarin Red S (Sigma Chemical, MO).

### Fourier Transform Infrared Spectroscopy (FTIR) analysis

MC cells were plated at a density of 4 × 10^5^ cells per 35 mm dish and cultured in α-minimum essential medium containing 10% FBS, 100 U/mL penicillin, and 100 μg/mL streptomycin. Upon confluence, cells were maintained in the mineralization medium (see above), and 75 ng/mL of BMP-2 with or without 8 µg/mL of LRR2-3 synthetic peptide and cultured for up to 2 weeks. MC cells were also cultured in the mineralization medium with or without LRR2-3 (i.e. no BMP-2 treatment) for up to 4 weeks. At 2 and 4 weeks of culture, the cell/matrix was washed three times with 0.2 M NH_4_HCO_3_ pH 8.0, collected, lyophilized, and subjected to FTIR analysis. Briefly, about 3 mg of each sample was mixed with 150 mg of KBr, finely ground and pressed into a thin pellet. FTIR spectra were collected using a Perkin Elmer Spectrum 400 spectrometer, with a resolution of 4 cm^−1^ and averaging 32 co-added scans. Unscrambler software (Camo, Norway) was used for spectral data analysis, which included second derivative processing of the spectra to narrow the broad absorbance peaks. The regions of interest were the amide I band for protein content (1700–1600 cm^−1^), the collagen band (1342–1332 cm^−1^), the ν_1_ PO_4_ band for mineral content (975–955 cm^−1^), the CO_3_ band reflecting carbonated incorporated into the mineral (888–868 cm^−1^), and the HPO_4_ band reflecting acid phosphate incorporated into mineral (1120–1115 cm^−1^). Second derivative spectral peak intensities reported are proportional to the amount of the protein and mineral components present in the cultures. Mineral crystallinity was estimated based on the ratio of the second derivative peak heights at ~1030 and 1020 cm^−1^ in the ν_3_ PO_4_ band. The peak assignments and analysis were based on well-established descriptions and procedures^32–34^.

### Molecular dynamic simulation

Using the X-ray crystal structure of BGN dimer (pdb ID; 2FT3^24^) as the starting template, we have modeled various peptides that were tested for their activities to enhance BMP-2 function, i.e. LRR2 (with a hinge), LRR3 (with a hinge), LRR1-2, LRR2-3, LRR1-3, LRR3-4 and LRR4-6. The peptide sequences are given in the Table 1. Each of these peptides is solvated, neutralized with appropriate amount of counter ions, and subjected to extensive MD calculations (of about 120 ns) after 10 ns equilibration simulations using the PMEMD module of the program suite Amber.14^35^. The force field required to represent proteins is Amber FF14SB, and explicit water molecules are represented by the TIP3P force field. The resultant solution structures are used for protein-protein docking to study the interactions of these peptides with BMP-2 and BMP-2 receptor (type IA and activin receptor type 2 A). The BMP-2 and BMP-2 receptor structures are from pdb IDs 1REW^36^ and 2GOO^30^ and the programs ZDOCK^37^ and FTDOCK^38^ were used for the protein-protein docking.

### Statistical analysis

All sample conditions were analyzed in triplicates and compared using One-way ANOVA with post-hoc Tukey-Kramer using InStat software (GraphPad). The data were presented as means ± S.E., and the differences were considered significant when P < 0.05.

## Electronic supplementary material

Dataset 1
