# Supplementary material for: Identification of the effector domain of biglycan that facilitates BMP-2 osteogenic function
Source: Sci Rep. 2018 May 4;8:7022. doi: 10.1038/s41598-018-25279-x (PMC5935668; doi:10.1038/s41598-018-25279-x)

**Identification of the effector domain of biglycan that facilitates BMP-2 osteogenic function**

**Prapaporn Jongwattanapisan^1,Ɨ^, Masahiko Terajima^2,Ɨ^, Patricia A. Miguez^3^, William Querido^4^, Hideaki Nagaoka^2^, Noriko Sumida^2^, Elizabeth Grace Gurysh^5^, Kristy M. Ainslie^5^, Nancy Pleshko^4^, Lalith Perera^6^, Mitsuo Yamauchi^2,*^**

^1^Department of Veterinary Medicine, Faculty of Veterinary Science, Chulalongkorn University, Bangkok, 10330, Thailand

^2^Oral and Craniofacial Health Sciences, School of Dentistry, University of North Carolina at Chapel Hill, Chapel Hill, NC-27599, USA

^3^Department of Operative Dentistry, Oral and Craniofacial Health Sciences, School of Dentistry, University of North Carolina at Chapel Hill, Chapel Hill, NC-27599, USA

^4^Department of Bioengineering, Temple University, Philadelphia, PA-19122, USA

^5^Division of Pharmacoengineering and Molecular Pharmaceutics, Eshelman School of Pharmacy, University of North Carolina at Chapel Hill, NC-27599, USA

^6^Genome Integrity and Structural Biology Laboratory, National Institute of Environmental Health Sciences, National Institutes of Health, Research Triangle Park, NC-27709, USA

**Supplemental Table S1.** Primers for generation of GST-BGN and GST-deletion constructs.

| GST-construct | Forward Primers | Reverse Primers |
| --- | --- | --- |
| BGN | 5’GCGGATCCGATGAGGAGGCTTCAGGT3’ | 5’GCCTCGAGCTACTTCTTATAATTTCC3’ |
| ΔNC | 5’GCGGATCCACACTGCTAGACCTG3 | 5’GCCTCGAGGGTGATGTTGTT3’ |
| N-LRR6 | Same as BG | 5’GCCTCGAGAGTGTGGCCTCTGA-3’ |
| LRR7-C | 5’GCGGATCCACCCTGAACGAACTT3’ | Same as BGN |

**Supplemental Table S2**. Physical and dynamical properties of the peptides used in MD simulations. (positive/negative means number of positively charged residues (such as lysine and arginine) and negatively charged residues (such as glutamate and aspartate) in each peptide.

| Peptide | Number of residues | Net charge (e) (positive/negative) | Averaged RMSD(Å)  over the last 20ns |
| --- | --- | --- | --- |
| LRR1-2 | 34 | -1(4/5) | 3.7 |
| LRR2-3 | 36 | 6(7/1) | 2.5 |
| LRR1-3 | 58 | 4(10/6) | 3.0 |
| LRR3-4 | 33 | 2(5/3) | 2.7 |
| LRR4-6 | 62 | 2(9/7) | 2.7 |
| LRR3 | 24 | 5(6/1) | 4.5 |
| LRR2 | 24 | 3(4/1) | 4.5 |

**Supplemental Figure S1. Full-length SDS-PAGE and Western blot images of GST-fused BGN constructs.** SDS-PAGE stained with Coommassie brilliant blue (a) and Western blot (WB) analyses with anti-GST antibody (b). Lane 1 and 4, ∆NC; lane 2 and 5, N-LRR6; lane 3 and 6, LRR7-C. The respective bands are indicated by red arrows. ∆NC, deletion constructs including core protein without N and C termini; N-LRR6, N terminal to leucine rich repeat domain (LRR)6; LRR7-C, LRR7 to C terminus.


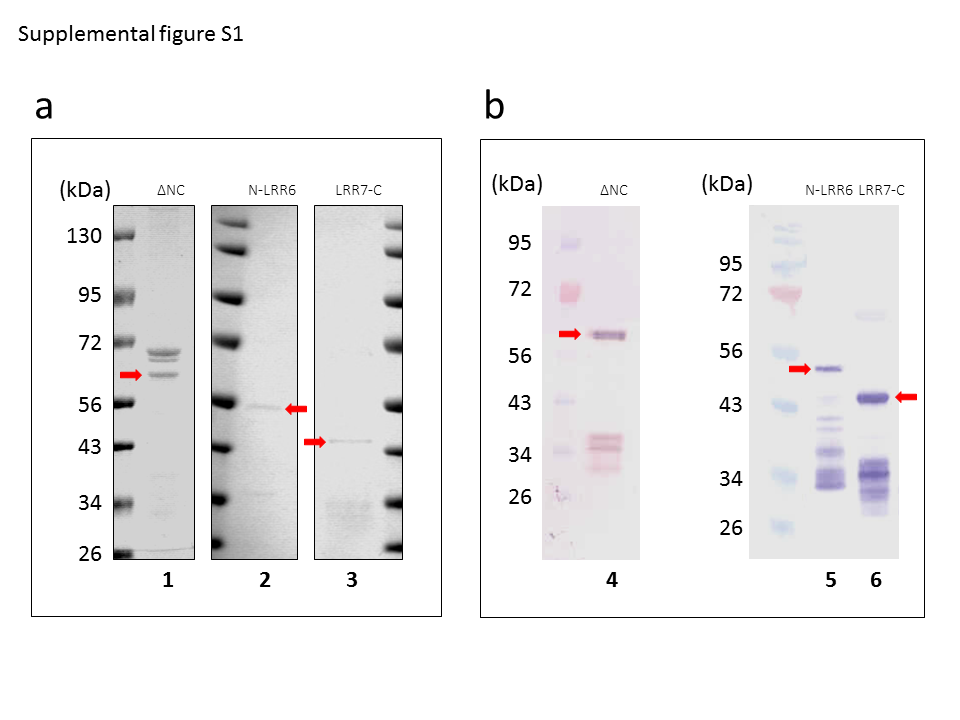


**Supplemental Figure S2. Western blot images of the effect of LRR2-3 on the BMP-2 induced signaling.** Western blot (WB) analyses with (a) anti-phospho-Smad1/5/9 antibody and (b) anti-Smad 1 antibody at 5, 15, 30, 120, 240 and 480 min.


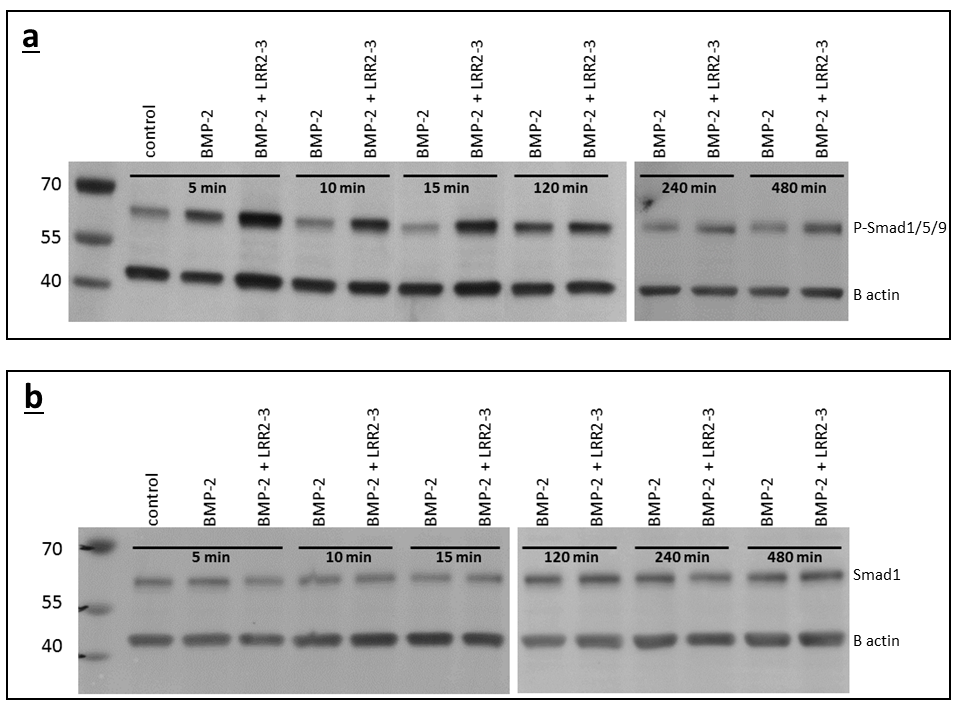

Supplement: Supplementary file 1 — Dataset 1 [file 41598_2018_25279_MOESM1_ESM.docx]
